# Supplementary material for: Early redox activities modulate Xenopus tail regeneration
Source: Nat Commun. 2018 Oct 16;9:4296. doi: 10.1038/s41467-018-06614-2 (PMC6191437; doi:10.1038/s41467-018-06614-2)
Supplement: Supplementary file 1 — Supplementary Information [file 41467_2018_6614_MOESM1_ESM.pdf]

Supplementary Information for Ferreira *et al.*

Early redox activities modulate *Xenopus* tail regeneration

Fernando Ferreira[1,2,*], VijayKrishna Raghunathan[3-5], Guillaume Luxardi[1], Kan Zhu[1] & Min Zhao[1,6,*]

(*Running title:* Integrating redox activities during regeneration)

[1]Department of Dermatology, Institute for Regenerative Cures, University of California, Davis, CA, USA.
[2]Departamento de Biologia, Centro de Biologia Molecular e Ambiental (CBMA), Universidade do Minho, Braga, Portugal.
[3]Department of Basic Sciences, University of Houston, TX, USA.
[4]The Ocular Surface Institute, College of Optometry, University of Houston, TX, USA.
[5]Department of Biomedical Engineering, Cullen College of Engineering, University of Houston, TX, USA.
[6]Department of Ophthalmology, Institute for Regenerative Cures, University of California, Davis, CA, USA.
*Corresponce: F.F., email: id3955@alunos.uminho.pt; M.Z., email: minzhao@ucdavis.edu.

Supplementary Figures

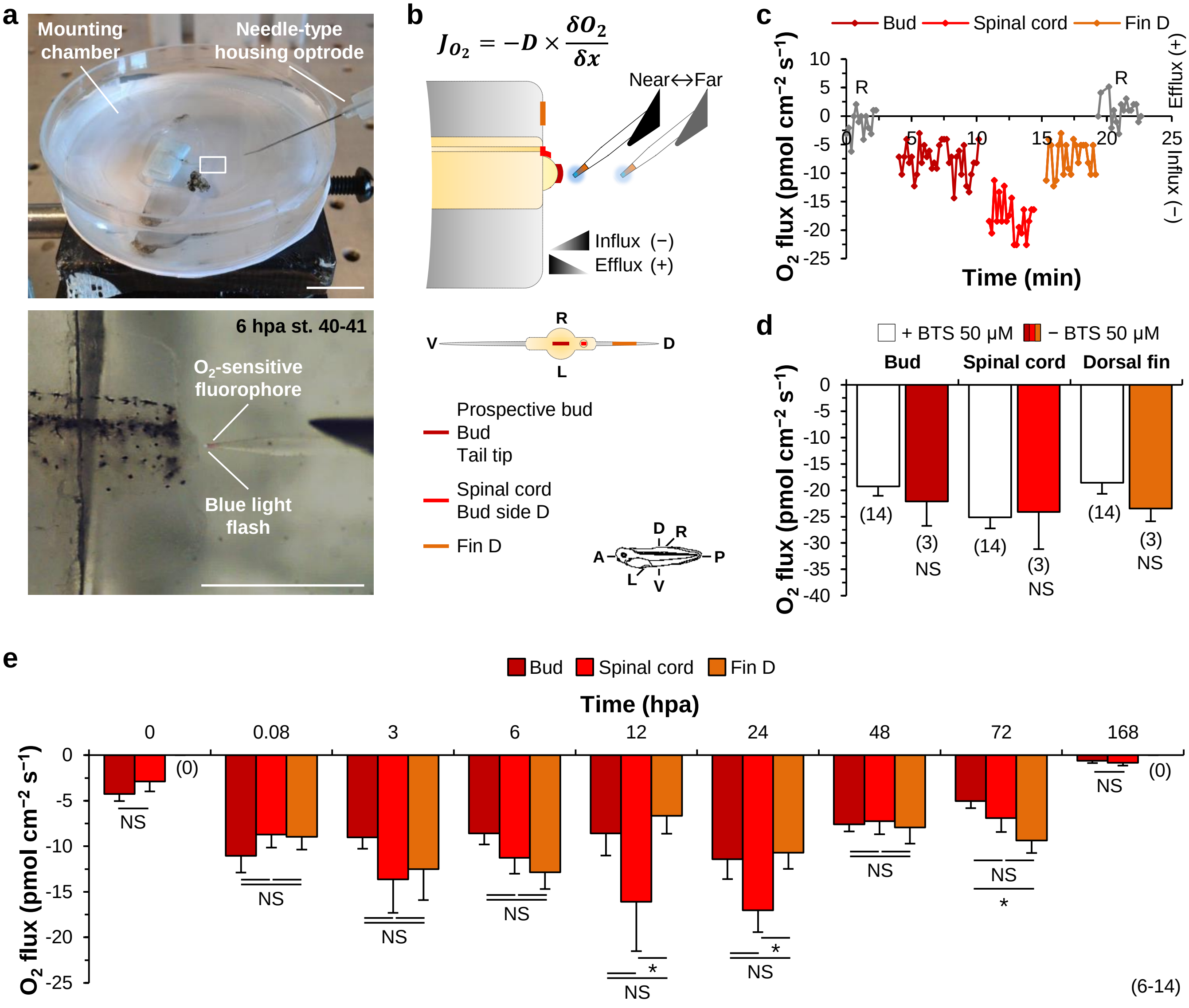

Supplementary Figure 1 | Spatiotemporal profile of extracellular O_2 flux during tail regeneration. (**a**,**b**) Experimental design, regions of interest (ROIs) and flux equation (Fick's first law detailed in Methods) for the O_2 flux profiling. Bottom panel in **a**: close up of rectangle in top panel. Scale bars: ~1 cm in top panel and ~1 mm in bottom panel. In **b**, sizes in schemes are relative approximations, except for the optrode that is zoomed in for detailed depiction of features labeled in **a**. ROIs were measured at the left-right (L/R) axis intersection (middle scheme (back view) in **b**). Ventral and dorsal fin measurements had similar fluxes, thus we just measured in the dorsal fin (annotated ROI). ROIs are color-coded and also apply to **c**-**e** and to subsequent figures. Photomicrograph and scheme of tails (lateral view) are displayed in the same orientation as the whole organism anteroposterior (A/P), dorsoventral (D/V) and L/R axes (bottom left scheme in **b**; applies to subsequent figures). (**c**) Representative result (MMR 0.1× 12 hpa). R: reference. (**d**) Chemical immobilization does not affect O_2 flux. Fluxes measured at 6 hpa in st. 45-46. Data of + BTS 50 μM are from Supplementary Fig. 3a, being siblings of part of the data of − BTS 50 μM; data of − BTS 50 μM are from a single batch of tadpoles. (**e**) Spatiotemporal profile of O_2 flux during regeneration in MMR 0.1× (control). Intriguingly, dorsal fins regenerated less efficiently than ventral ones (biased fin regeneration) and non-regenerated

dorsal fins had larger O_2 influxes than regenerated dorsal (and ventral) ones at 72 hpa. Since the profiling is to elucidate regeneration, we excluded the non-regenerated dorsal fin data at 72 hpa (3 out of 9). Statistical analyses were performed by unpaired Student's *t*-test (two-tailed *p* value). Data are presented as mean±s.e.m. *n* biological replicates indicated in brackets. NS, non-significant; *, $p<0.05$.

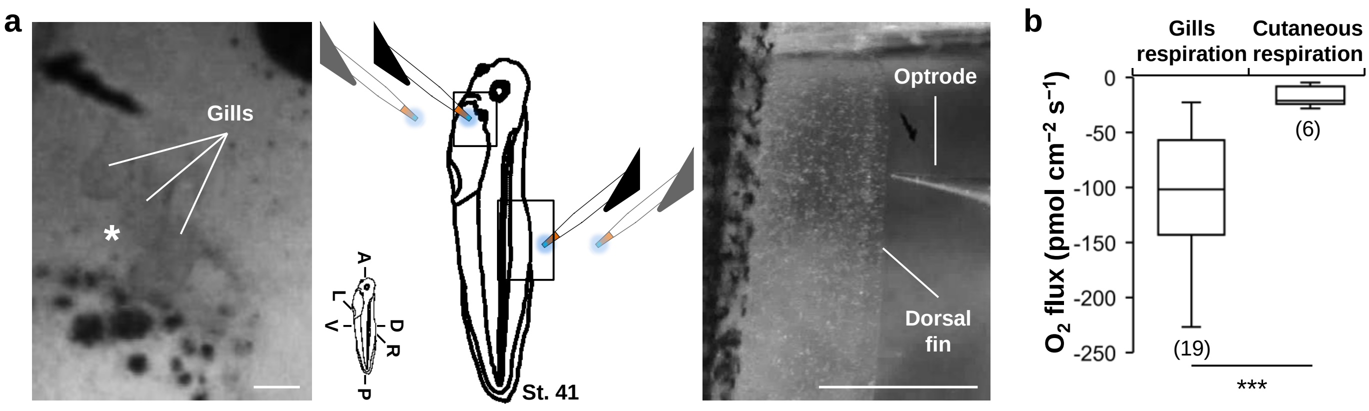

Supplementary Figure 2 | Modalities of respiration in tadpoles. (**a**,**b**) Tadpoles uptake O_2 through gills and cutaneous surfaces with different magnitudes. (**a**) Center: Experimental design. Right and left panels: representative gills and dorsal fin close ups of rectangles in center. Gill measurements were taken in the middle gill of left axis (white *). Scale bars: ~100 µm in right panel and ~0.5 mm in left panel. (**b**) O_2 influx in the two respiration surfaces (in MMR 0.1×). Data of cutaneous respiration are from Supplementary Fig. 4e, being siblings of part of the gills respiration data. Statistical analyses were performed by unpaired Student's *t*-test (two-tailed *p* value). Data are presented as median±min to max (with outliers). *n* biological replicates indicated in brackets. ***, $p<0.001$.

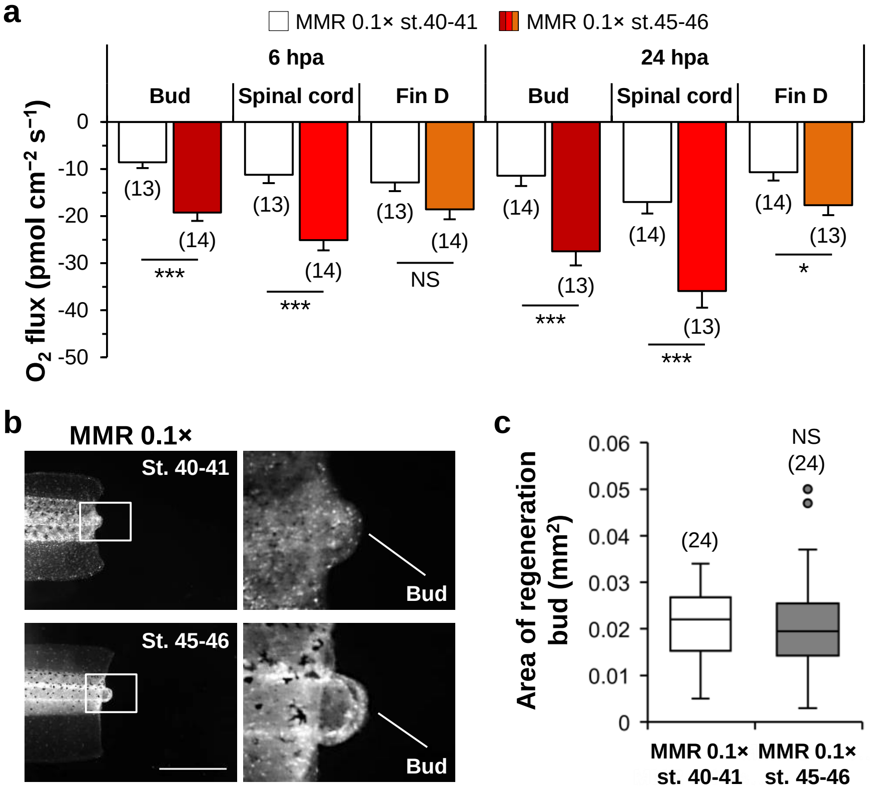

Supplementary Figure 3 | O_2 influx correlation with regeneration is independent of bud size. (**a**) Spatial profile of O_2 flux measured in two different time-points in tails amputated in regenerative or refractory periods. Spatial ROIs have increased influxes in tails amputated in the refractory period. The absent (at 6 hpa) or lower (at 24 hpa) disparities of O_2 influx in fins point to a more bud-specific and so regeneration-specific O_2 flux. Data of regeneration bud are the same as those shown in Fig. 2c. (**b**,**c**) O_2 influx magnitude is independent of bud size at 6 hpa in tails amputated in regenerative or refractory periods. Nonetheless, buds are apparently different in morphology and likely different in function (do not drive regeneration in refractory period). (**b**) Representative buds at 6 hpa. Left panels: close up of correspondent rectangles in right panels. Scale bar: 1 mm. (**c**) Area of buds at 6 hpa. Overall tadpole size is lower in st. 40-41 than in 45-46[1], but since buds have similar sizes, thereby overall size could only affect fin flux. We did not test this directly, *i.e.*, we did not measured intact *vs.* regenerating fins at same age. Theoretically, larger tadpoles should have lower O_2 influx in intact fins (cutaneous respiration; Supplementary Fig. 2), due to increased surface area to volume ratio (ratio and the O_2 uptake are, in principle, inversely proportional). In fact, fully regenerated tails at 7 dpa (larger tadpoles) have a 7-fold reduction in O_2 influx compared to tails before amputation (smaller tadpoles; Fig. 1). As noted elsewhere (Supplementary Fig. 1e), non-regenerating fins presented higher O_2 influxes in regenerative period at 72 hpa; this (and not the overall size) agrees with the elevated O_2 influx at 24 hpa in refractory period non-regenerating fins compared with regenerating fins. The differences between regenerating and non-regenerating epithelia, might not explain the disparate O_2 fluxes. This is because refractory epithelium is thicker[2-4] and thus possibly less permeable to O_2. Thereby, mechanism remains elusive. Statistical analyses were performed by unpaired Student's *t*-test (two-tailed *p* value). Data are presented as mean±s.e.m. (**a**), or median±min to max (with outliers) (**c**). *n* biological replicates indicated in brackets. NS, non-significant; *, $p<0.05$; ***, $p<0.001$.

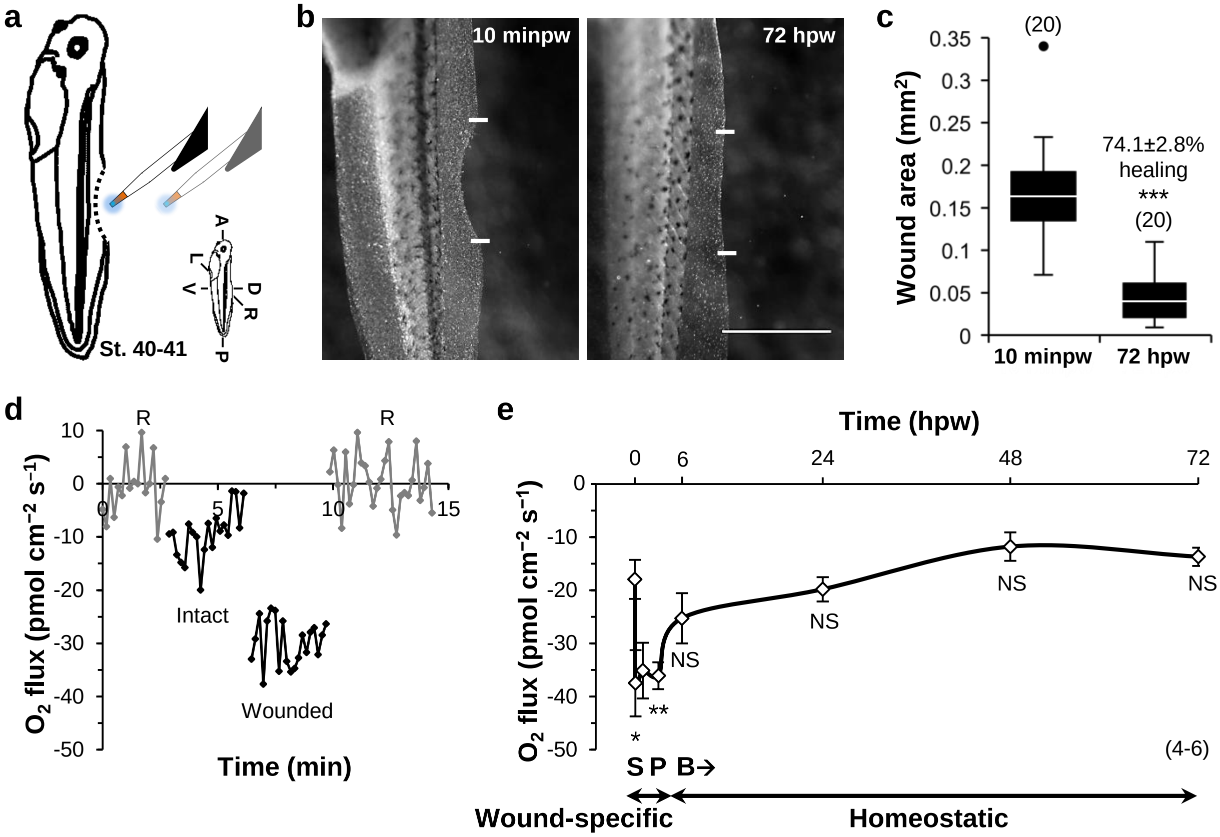

Supplementary Figure 4 | Temporal profile of extracellular O_2 flux during wound healing. (**a**) Experimental design. (**b**) Representative dorsal fins after wounding (10 min) and healing (72 h) (in DMSO 0.1%). White solid lines: wound edges; scale bar: 1 mm. (**c**) Area of unhealed and percentage of healed fin. After 72 hpw there was no significant fin healing (72 hpw: 0.055±0.016 mm^2; 120 hpw: 0.054±0.018 mm^2; n=5, p=0.968). (**d**) Representative result (MMR 0.1× 0 and 5 minpw). (**e**) Temporal profile of O_2 flux during wound healing (in MMR 0.1×). Profile is descriptively divided into three parts: S, slope – instantaneous; P, plateau – until 3 hpw; and B, baseline – after 3 hpw; comprising two phases: wound-specific and homeostatic. Statistical analyses were performed by unpaired Student's t-test (two-tailed p value). Data are presented as median±min to max (with outliers) (**c**), or mean±s.e.m. (**e**). n biological replicates indicated in brackets. NS, non-significant; *, p<0.05; **, p<0.01; ***, p<0.001.

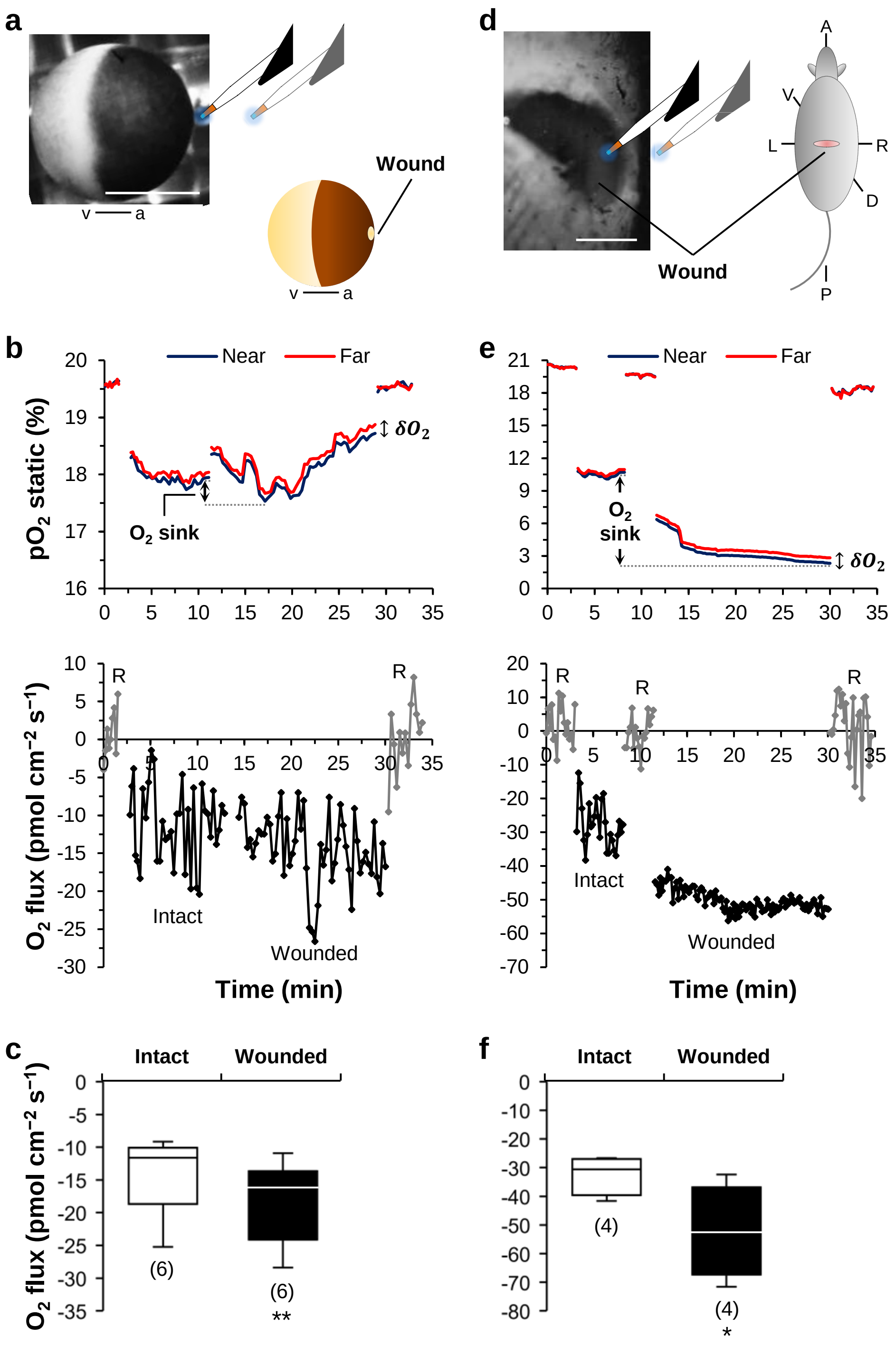

Supplementary Figure 5 | O_2 sink is an instantaneous and conserved response to injury. (**a**,**b**) Experimental designs. a: animal pole; v: vegetal pole. Scale bars: ~0.25 mm in **a** and ~1 mm in **b**. Sizes in oocyte (lateral view) and mouse (top view) schemes are relative approximations. (**b**,**e**) Representative results (oocyte: MMR 1×; mouse skin: PBS). Top plots: O_2 static measurements at near and far poles; bottom plots: O_2 flux calculated using the difference of near and far poles values (δO_2) from top plots (after conversion to O_2 concentration as detailed in Methods). Injury-induced peak O_2 sinks – *i.e.*, minimum intact level minus maximum wounded level – are annotated: 0.4% for oocyte and 8.4% for mouse skin. Averages of peak O_2 sinks were as follows: 0.3±0.1% for oocytes (n=6) and 7.3±2.1% for mice skin (n=4). Being unicellular, oocytes might have intracellular and extracellular pO_2

closer to equilibrium. Therefore, the O_2 gradient established upon wounding is much smaller in oocytes than in mice skin, generating a much less penetrant O_2 sink. Reference (R), intact and wounded measurements (labels in bottom plots) are delimited by a discontinuity in the solid lines. *x* axis title in bottom plots applies to top plots; *y* axis titles in **b** apply to **e**. (**c**,**f**) O_2 influxes elevate upon wounding in oocytes (29% increase; p=0.003) and in mice skin (61% increase; p=0.033). Statistical analyses were performed by paired Student's *t*-test (two-tailed *p* value for **c**, or one-tailed *p* value for **f**). Data are presented as median±min to max (with outliers). *n* biological replicates indicated in brackets. *, $p<0.05$; **, $p<0.01$.

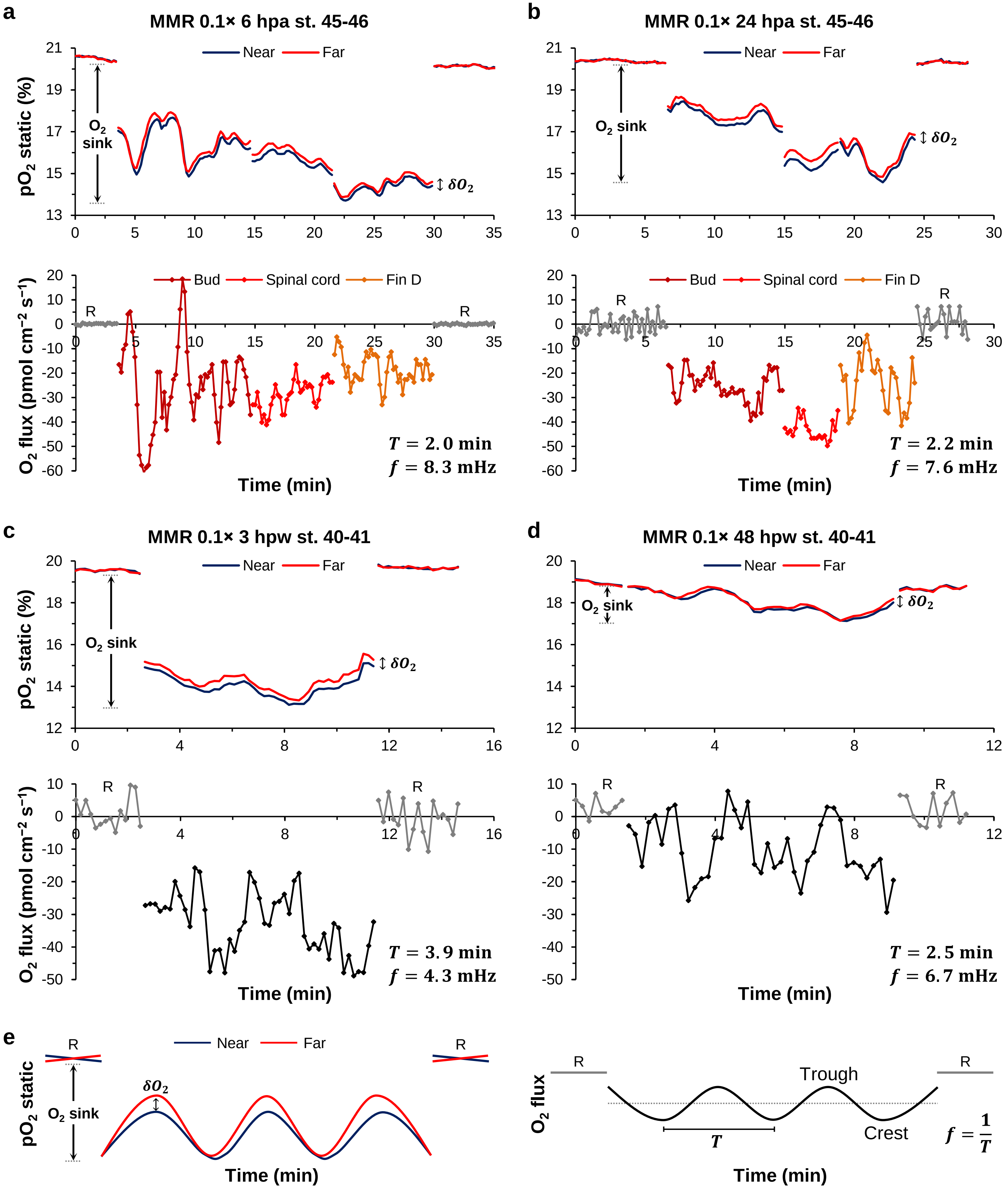

Supplementary Figure 6 | O_2 oscillations. (**a-d**) Representative O_2 oscillations and oscillatory plateaus in amputated tails (**a**,**b**) and wounded fins (**c**,**d**). Top plots: O_2 static measurements at near and

and far poles; bottom plots: O_2 flux calculated using the difference of near and far poles values (δO_2) from top plots (after conversion to O_2 concentration as detailed in Methods). References (R) do not appear to present oscillations, excluding noise and drift as causes for the measured oscillations. Reference and ROI measurements (legend and label in bottom plots) are delimited by a discontinuity in the solid lines. *x* axis title in bottom plots applies to top plots; *y* axis titles in **a**/**c** apply to **b**/**d**. Injury-specific peak O_2 sinks – *i.e.*, minimum reference level minus maximum specimen level – are annotated. O_2 sink drastically decreases with fin healing in **d**; same occurs during regeneration as decreased O_2 influx proves (Fig. 1b,c). (**e**) Schematic plots depicting the apparently more common oscillation profile observed in **a**-**d** and in all other recordings acquired. Right plot: O_2 static at near and far poles; left plot: O_2 flux calculated from right plot (after conversion to O_2 concentration). The oscillation profile seems counter-intuitive, because when O_2 sink is larger O_2 flux is smaller and *vice-versa*. Thereby, O_2 static and O_2 flux oscillations (waves) are in antiphase, *i.e.*, crests in left plot are troughs in right plot and *vice-versa*. Other apparently less common oscillation profiles were also note. More exhaustive and robust qualitative and quantitative analyses of oscillations and putative oscillations were not performed. Such analyses would have required a specific experimental design. The most important design requirement would have been the increase of sampling time (*e.g.*, to 30 min per ROI) to be able to encompass more oscillation cycles (periods) and to clearly identify potential false positives and false negatives. As described in Methods, we recorded the fluxes usually for 2-5 min per ROI; as the representative oscillations attest, 2 min is a probable minimal threshold to detect a single period, therefore many plots could not be analyzed to robustly study oscillations. For these reasons, we avoided to calculate means±SEMs and instead selected cases that seem to represent the range of the reasonably identifiable oscillations. Thereby, for the period, the range seems to be around 2-4 min and for the frequency, the range seems to be around 4-8 mHz. Equation: frequency (f) is the multiplicative inverse (reciprocal) of the period (T) and *vice-versa*. Annotated periods and frequencies were calculated by averaging most or all crest-to-crest and trough-to-trough times for each plot (*e.g.*, the 3 crests and 3 troughs in bottom **d** provided 4 peak-to-peak time values that when averaged gave a period of 2.5 min and a frequency of 6.7 mHz).

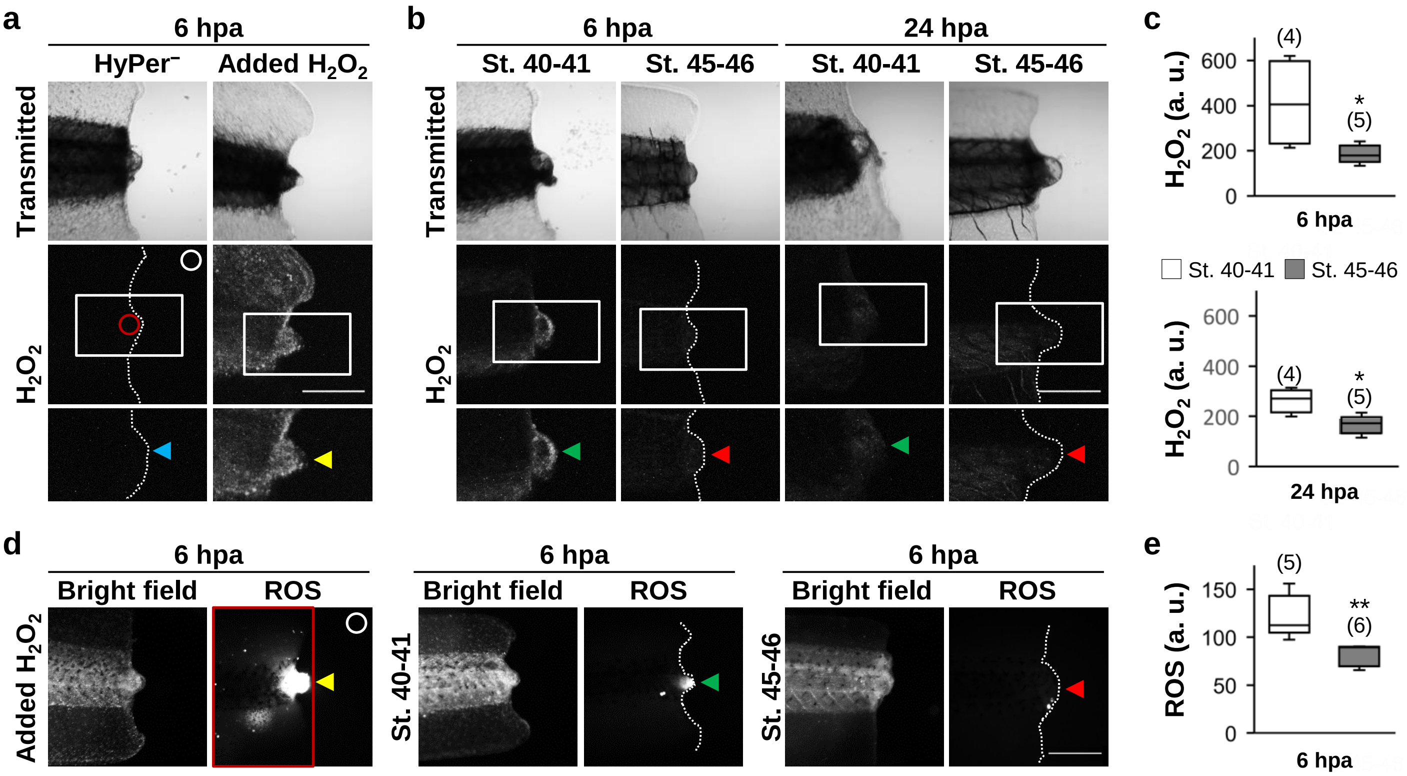

Supplementary Figure 7 | Refractory period tadpoles have lower ROS/H_2O_2 levels in the bud. (**a**) Controls validating the measurement of H_2O_2 using HyPer transgenic tadpoles. Representative tails under transmitted light (top panels) and fluorescence imaging (488/515 excitation/emission nm; middle and bottom panels) (applies to **b**). Transgenesis' negative control (*i.e.*, no HyPer expression; left panels) was devoid of any measurable fluorescence (blue arrowhead) and experimental positive control (H_2O_2 1 mM added to bath for 15 min; right panels) had very strong fluorescence (yellow arrowhead), validating the measurement system. Several tadpoles presented similar readouts at both 6 and 24 hpa in st. 40-41. (**b**,**c**) HyPer transgenic tadpoles during different regenerative periods. (**b**) Representative tails in regenerative (st. 40-41) and refractory (st. 45-46) periods at 6 and 24 hpa. Bottom panels: close up of corresponding rectangles in middle panels. (**c**) Semi-quantitative analysis of H_2O_2 levels (background subtracted; white circular ROI in **a**) in both conditions in the bud (red circular ROI in **a**) at 6 and 24 hpa. Refractory period H_2O_2 levels are 122 and 59% lower than regenerative levels at 6 and 24 hpa, respectively (p=0.032 in both). Box and whiskers plots legend applies to **e**. (**d**,**e**) Secondary validation of HyPer results using the ROS-sensitive dye CM-H_2DCFDA in wild-type tadpoles. (**d**) Representative tails under bright field (left panels) and fluorescence imaging (GFP channel; right panels) in regenerative (st. 40-41) and refractory (st. 45-46) periods at 6 hpa. Yellow arrowhead: very high ROS levels in the prospective regeneration bud; green arrowhead: high ROS/H_2O_2 levels in the (prospective) bud; red arrowhead: low ROS/H_2O_2 levels in the (prospective) bud; scale bars: 0.5 mm. (**e**) Semi-quantitative analysis of ROS levels (background subtracted; white circular ROI in **c**) in both conditions in the whole imaged tail (red rectangular ROI in **c**) at 6 hpa. Refractory period ROS levels are 46% lower than regenerative levels at 6 hpa (p=0.004). a. u.: arbitrary units. Statistical analyses were performed by non-parametric Mann Whitney test. Data are presented as median±min to max (with outliers). *n* biological replicates indicated in brackets. *, p<0.05; **, p<0.01.

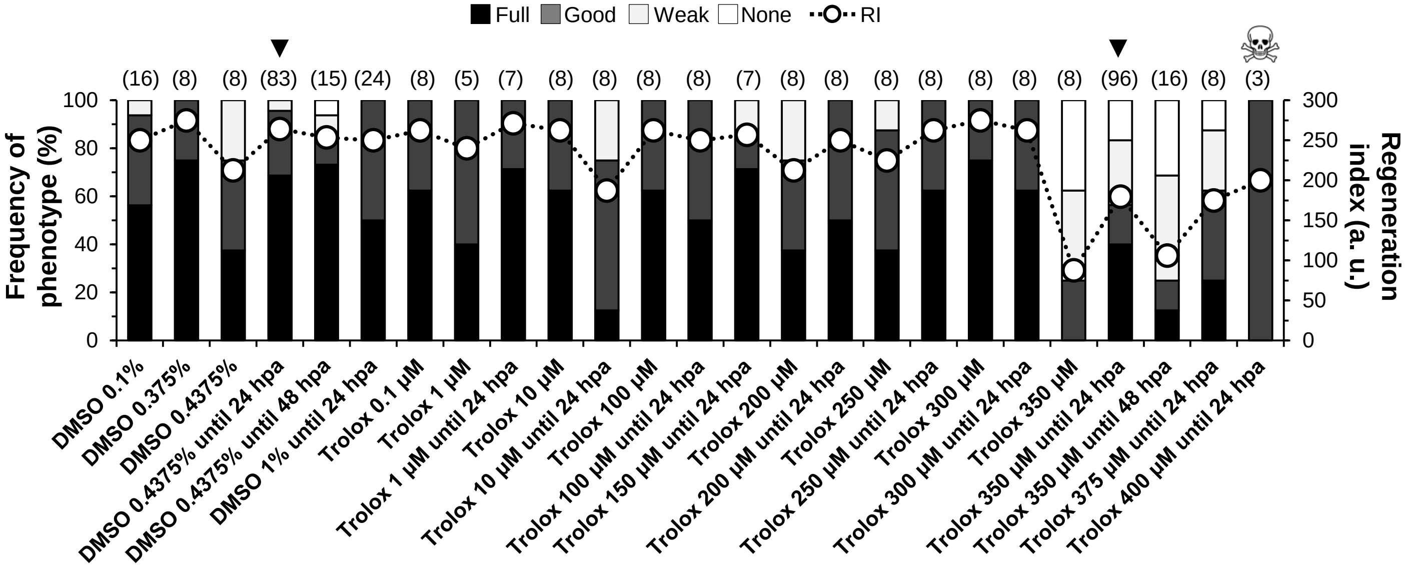

Supplementary Figure 8 | Dose-exposure screening to fine-tune pharmacological treatments during regeneration: trolox. Selection was based on the maximal penetrance with minimal observable toxicity and developmental side defects. DPI dose-exposure screening was previously performed[5]. Black arrowheads: selected dose-exposure and respective vehicle-control. Skull and crossbones symbol: mortality >50%. RI: regeneration index; a. u.: arbitrary units. *n* biological replicates indicated in brackets.

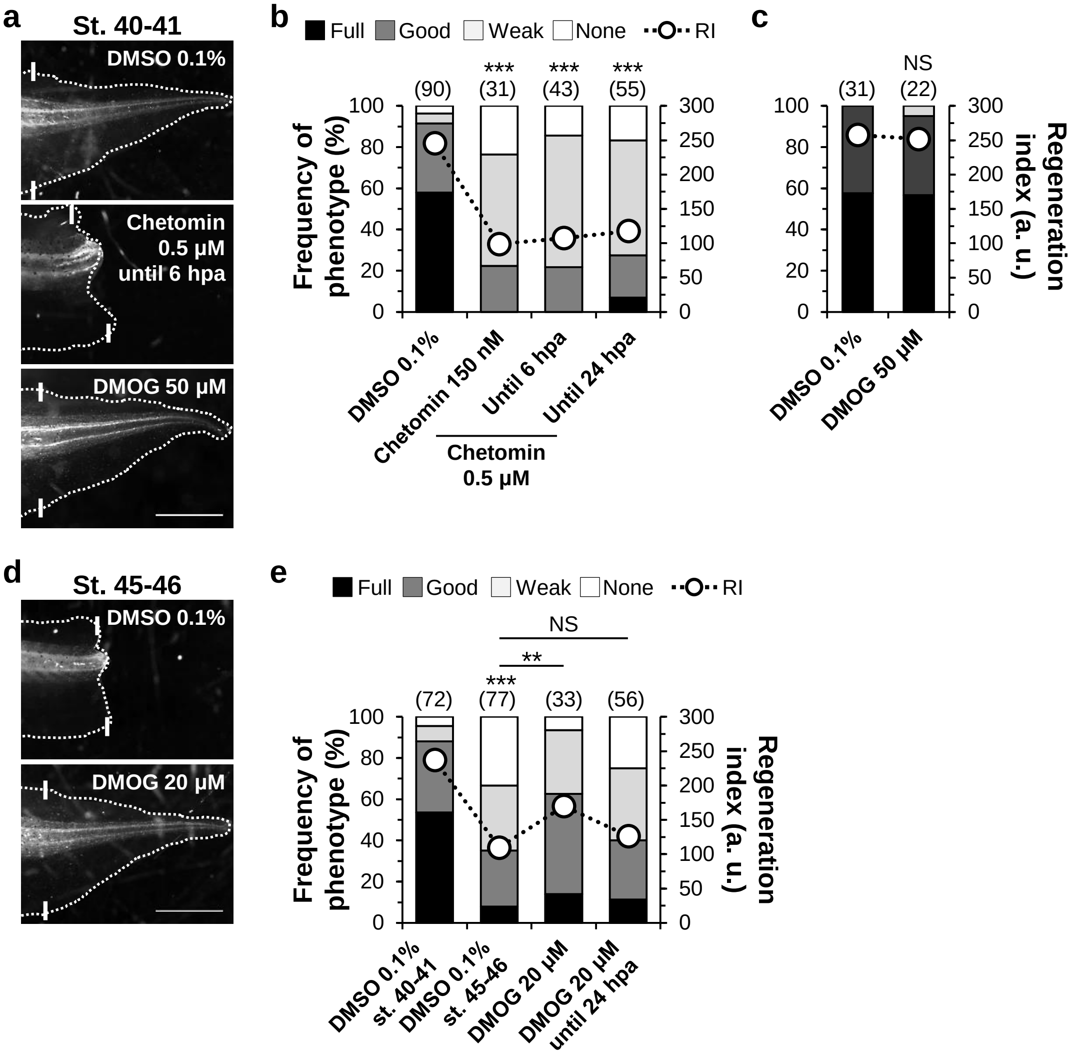

Supplementary Figure 9 | HIF-1α is necessary for and sufficient to induce regeneration. (**a**,**b**) Alternative HIF-1α inhibitor chetomin impairs regeneration and short-term exposure (until 6 or 24 hpa) to it is similarly penetrant in the impairment. (**a**) Representative tails at 7 dpa in vehicle-control and pharmacological treatment from tadpoles amputated at st. 40-41. (**b**) Qualitative and quantitative analyses of regeneration efficiency for the different conditions tested. (**a**,**c**) Drug-enhanced HIF-1α stabilization does not affect regeneration in tadpoles amputated at st. 40-41. (**c**) Qualitative and quantitative analyses of regeneration efficiency for the different conditions tested. Primary vertical axis title in **b** also apply to **c**; secondary vertical axis title in **c** also apply to **b**; stacked bars legend in **b** applies to **c**. (**d**,**e**) Short-term exposure (until 24 hpa) to DMOG is not sufficient to induce regeneration, despite a slight induction. (**d**) Representative tails at 7 dpa in vehicle-control and pharmacological treatment from tadpoles amputated in refractory period. (**e**) Qualitative and quantitative analyses of regeneration efficiency for the different conditions tested. Data of DMSO 0.1% st. 40-41 and st. 45-46 and DMOG 20 µM are the same as those shown in Fig. 4f, being siblings of the data of DMOG 20 µM until 24 hpa. White solid lines: amputation plane; scale bars: 1 mm. RI: regeneration index; a. u.: arbitrary units. Statistical analyses were performed by Fisher's exact test (two-tailed p value). n biological replicates indicated in brackets. NS, non-significant; **, $p<0.01$; ***, $p<0.001$.

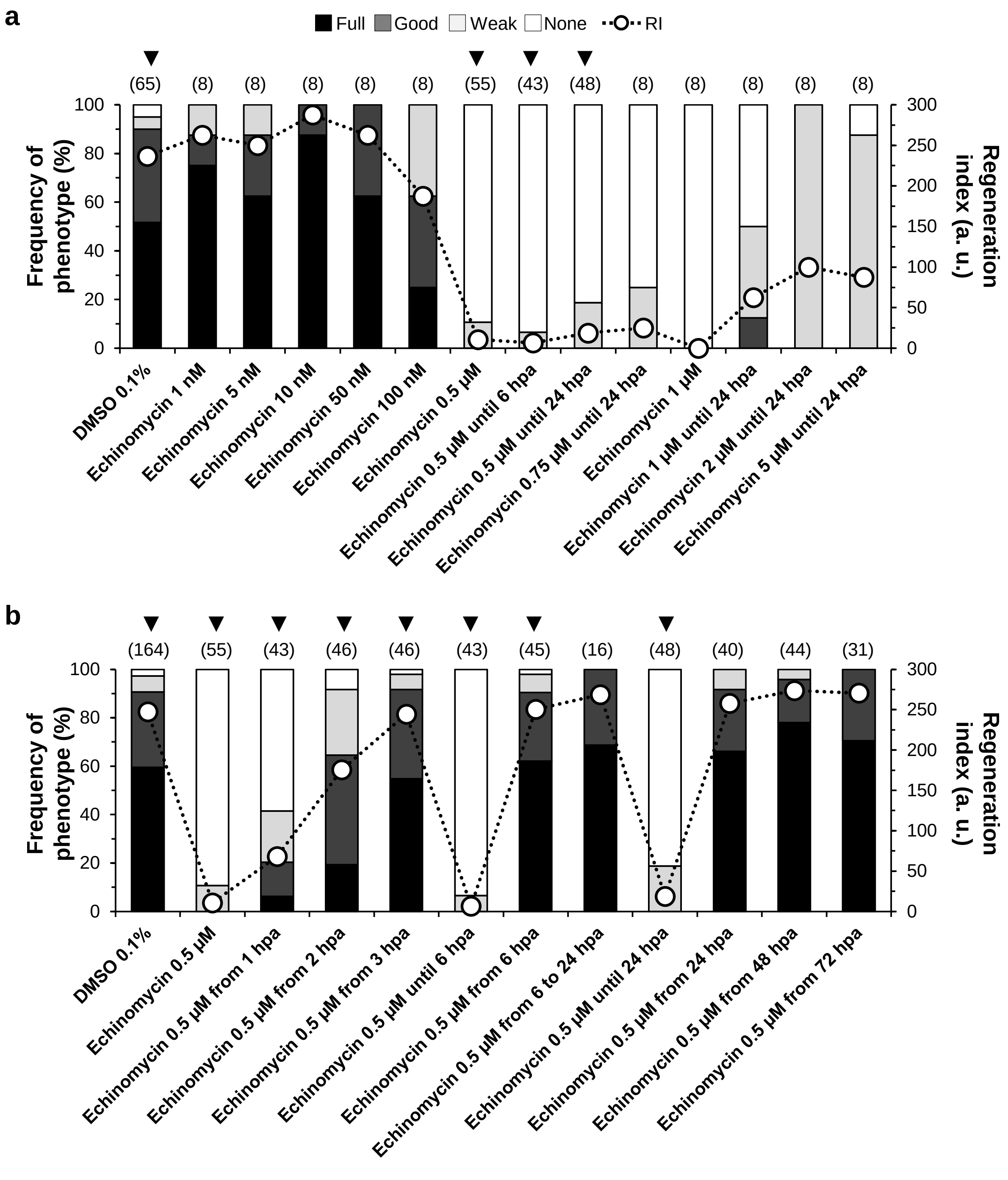

Supplementary Figure 10 | Dose-exposure screening to fine-tune pharmacological treatments during regeneration: echinomycin. (**a**) Gross screening. (**b**) Refined screening. Selection was based on the maximal penetrance with minimal observable toxicity and developmental side defects. Black arrowheads: selected dose-exposures and respective vehicle-control. RI: regeneration index; a. u.: arbitrary units. *n* biological replicates indicated in brackets.

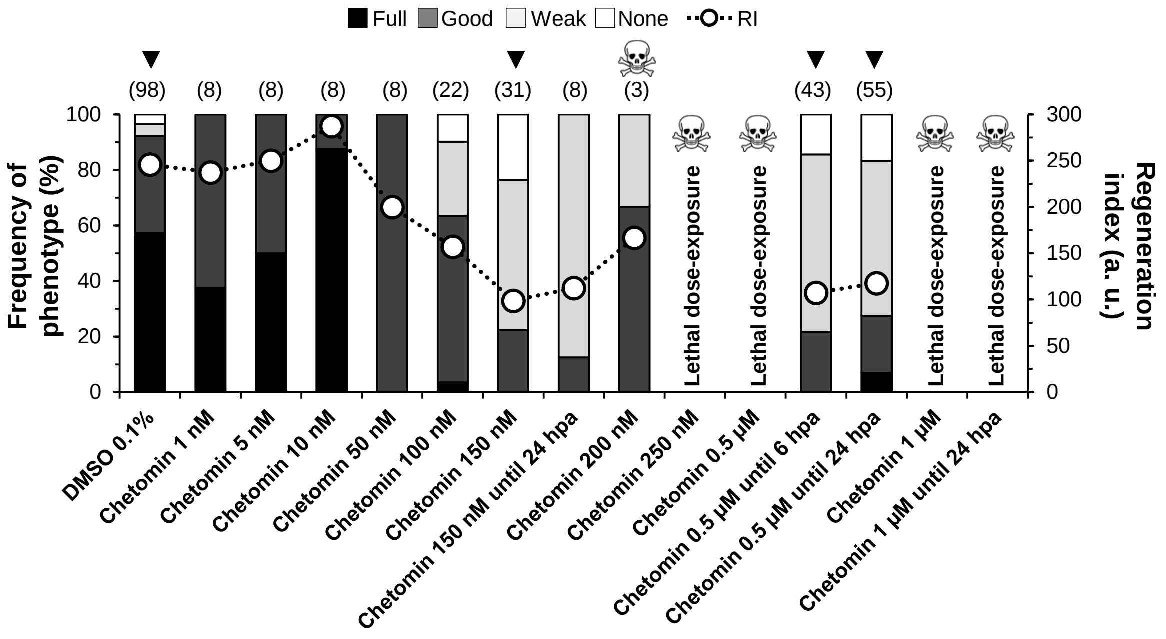

Supplementary Figure 11 | Dose-exposure screening to fine-tune pharmacological treatments during regeneration: chetomin. Selection was based on the maximal penetrance with minimal observable toxicity and developmental side defects. Black arrowheads: selected dose-exposures and respective vehicle-control. Skull and crossbones symbol: mortality >50%. RI: regeneration index; a. u.: arbitrary units. *n* biological replicates indicated in brackets.

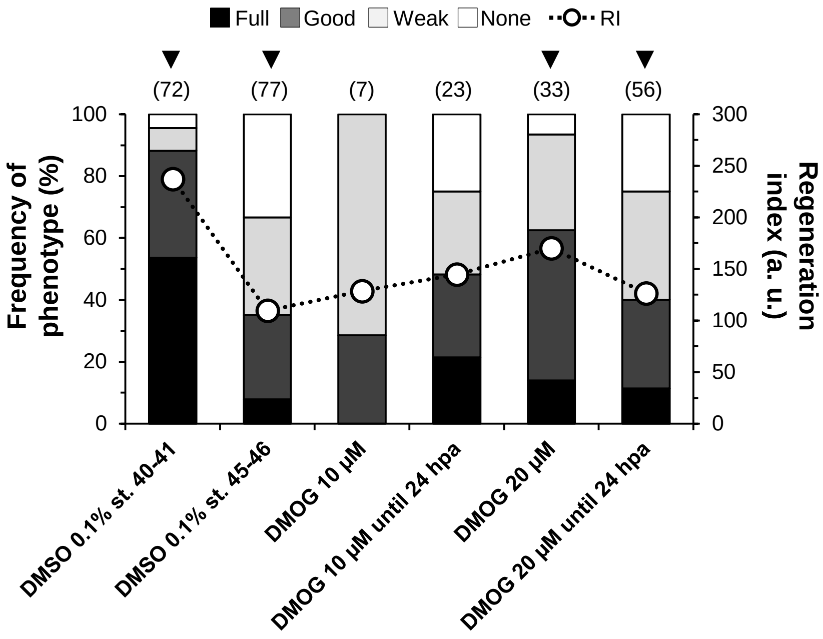

Supplementary Figure 12 | Dose-exposure screening to fine-tune pharmacological treatments during regeneration: DMOG. Selection was based on the maximal penetrance with minimal observable toxicity and developmental side defects. Black arrowheads: selected dose-exposures and respective vehicle-controls. RI: regeneration index; a. u.: arbitrary units. *n* biological replicates indicated in brackets.

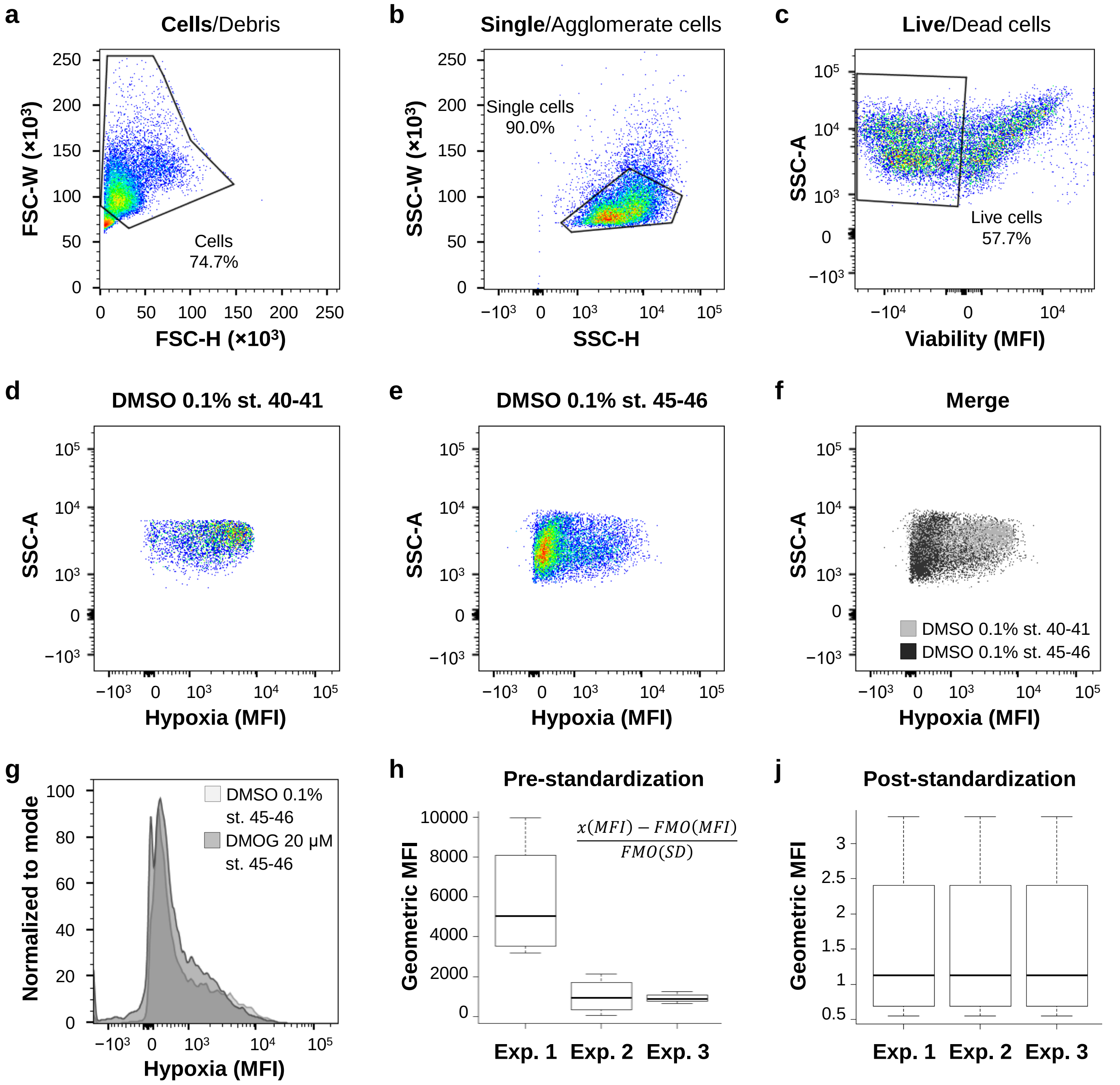

Supplementary Figure 13 | Stepwise data analysis of flow cytometry. (**a-c**) Representative flow cytograms of sequential gating. Debris (**a**), cell fragments and agglomerates (**b**), and dead cells (Aqua blue positive) (**c**) were excluded to gate live single cells. (**d-f**) Post-gating representative flow cytograms of cells analyzed in the fluorescein (FITC) channel. Merged data in **f** is the same as that shown in the bottom panel of Fig. 5a, presented as discriminated cells instead of histogram. FSC: forward scatter pulse; SSC: side scatter pulse; W: width; H: height; A: area; MFI: mean fluorescence intensity. (**g**) HIF-1α stabilizing drug does not induce hypoxia in the refractory period at 1 hpa. (**h**,**j**) Standardization of independent flow cytometry experiments for robust cross comparison. Inset in **h**: standardization equation (details in Methods). Distribution of fluorescence intensity per experiment pre- (**h**) and post-standardization (**j**). Exp.: experiment.

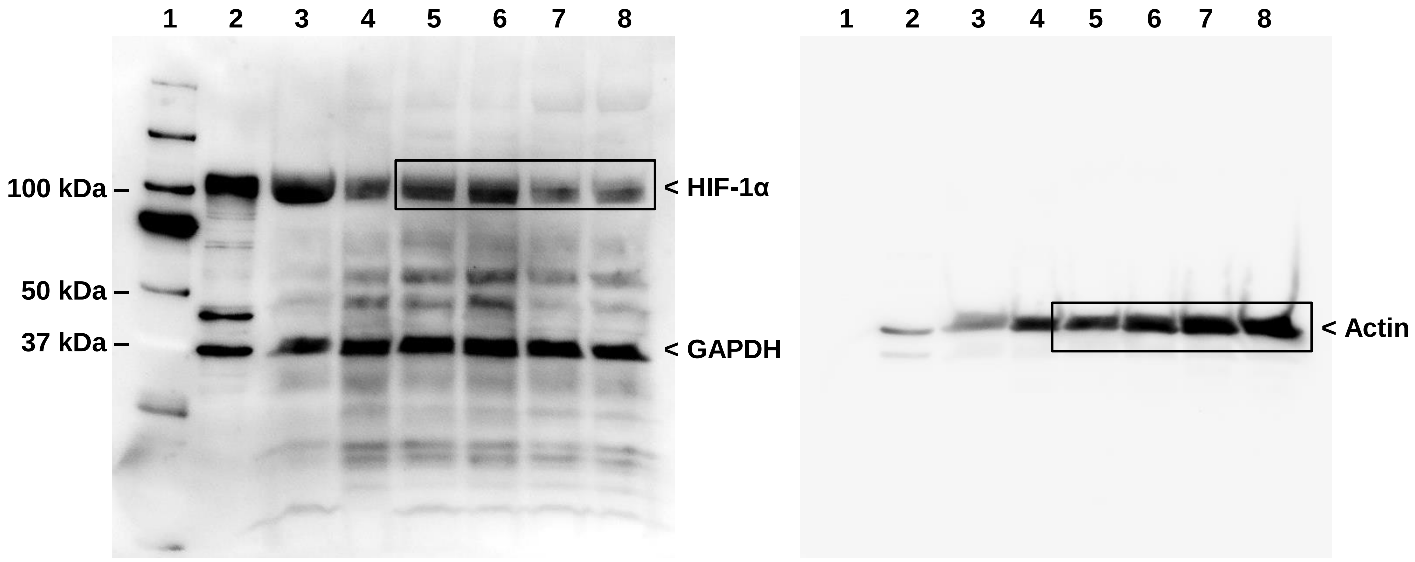

Lanes

1. Ladder
2. HIF-1α lysate (5 μg)
3. DMSO 0.1% st. 40-41, 1 huncut (whole tadpole)
4. DMSO 0.1% st. 45-46, 1 huncut (whole tadpole)
5. DMSO 0.1% st. 40-41, 1 hpa
6. DPI 1 μM st. 40-41, 1 hpa
7. DMSO 0.1% st. 45-46, 1 hpa
8. DMOG 20 μM st. 45-46, 1 hpa

Supplementary Figure 14 | Uncropped membranes of Western blotting against HIF-1α. Black rectangles mark the cropped membrane used in Fig. 5. Technical positive control (HIF-1α lysate in lane 2) validates selected antibody and respective specificity. GAPDH was initially used as reference protein; however, later reblotting of the membranes with actin antibody (right membrane) revealed less variability among lanes.

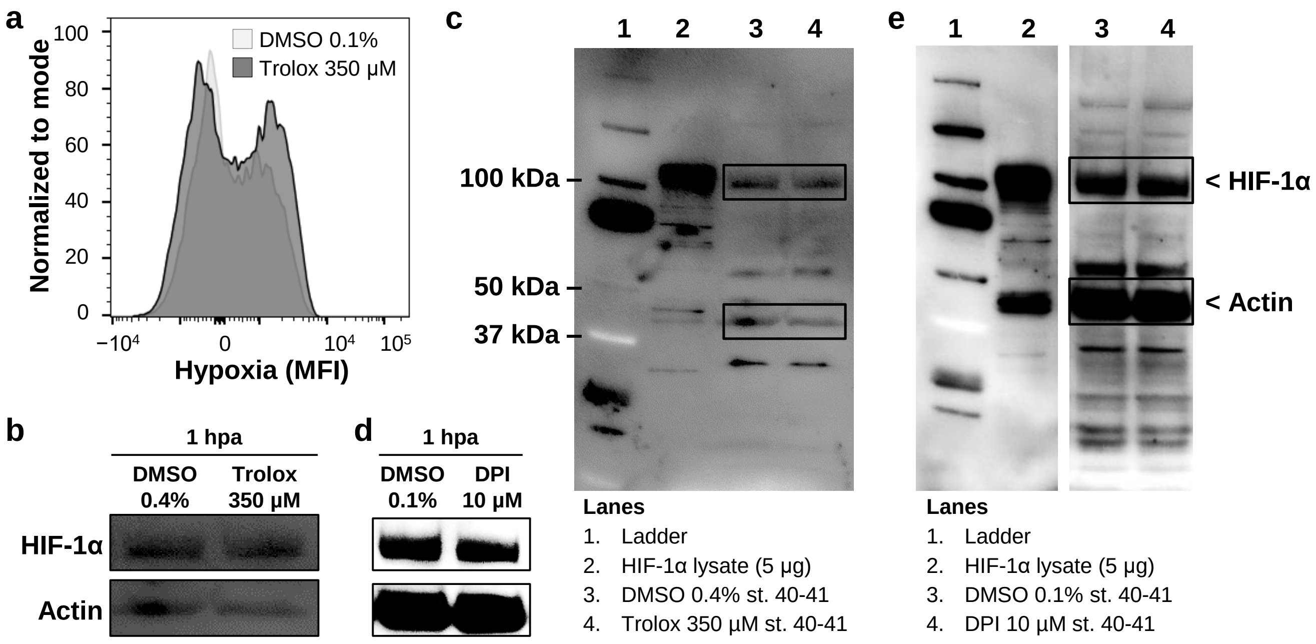

Supplementary Figure 15 | ROS *per se* neither affect hypoxia nor stabilize HIF-1α. Flow cytogram (**a**) and Western blotting (**b**) of DMSO 0.1%/0.4% st. 40-41 *vs.* trolox 350 µM st. 40-41 at 1 hpa. MFI: mean fluorescence intensity. (**d**) Western blotting showing that a ten-fold increase in the inhibition of ROS production does not proportionally decrease HIF-1α stability levels. This modulation of ROS production and scavenging implies that ROS do not directly stabilize HIF-1α. (**c**,**e**) Uncropped membrane. Black rectangles mark the cropped membrane used in **b** and **d**, respectively. Technical positive control (HIF-1α lysate) in lanes 2.

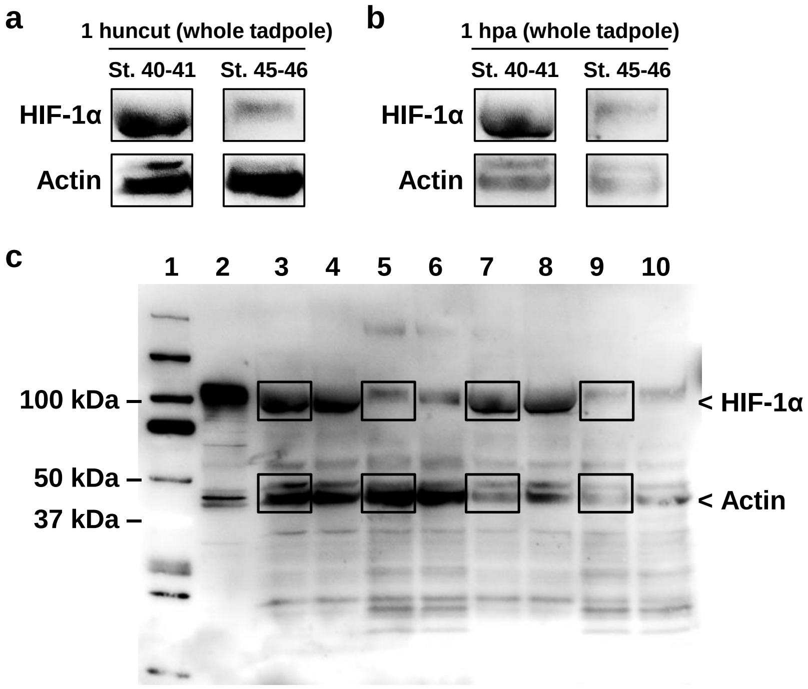

Lanes

1. Ladder
2. HIF-1α lysate (5 µg)
3. DMSO 0.1% st. 40-41, 1 huncut (whole tadpole)
4. DPI 1 µM st. 40-41, 1 huncut (whole tadpole)
5. DMSO 0.1% st. 45-46, 1 huncut (whole tadpole)
6. DMOG 20 µM st. 45-46, 1 huncut (whole tadpole)
7. DMSO 0.1% st. 40-41, 1 hpa (whole tadpole)
8. DPI 1 µM st. 40-41, 1 hpa (whole tadpole)
9. DMSO 0.1% st. 45-46, 1 hpa (whole tadpole)
10. DMOG 20 µM st. 45-46, 1 hpa (whole tadpole)

Supplementary Figure 16 | Age-dependent (stage-specific) HIF-1α stabilization predicts regeneration efficiency. (**a**-**c**) Western blotting against HIF-1α. HIF-1α stability levels are far higher in regenerative (st. 40-41) than in refractory (st. 45-46) period tadpoles. Differential trend is similar in both uncut (**a**) and amputated (**b**) whole tadpoles. Independent Western blots gave consistent readouts; *e.g.*, compare lanes 3 *vs.* 4 in Supplementary Fig. 14. Other, unstudied, developmental or physiological effects of HIF-1α stability disparity in the intriguing refractory period warrant future research. (**c**) Uncropped membrane. Black rectangles mark the cropped membrane used in **a** and **b**. Technical positive control (HIF-1α lysate) in lane 2.

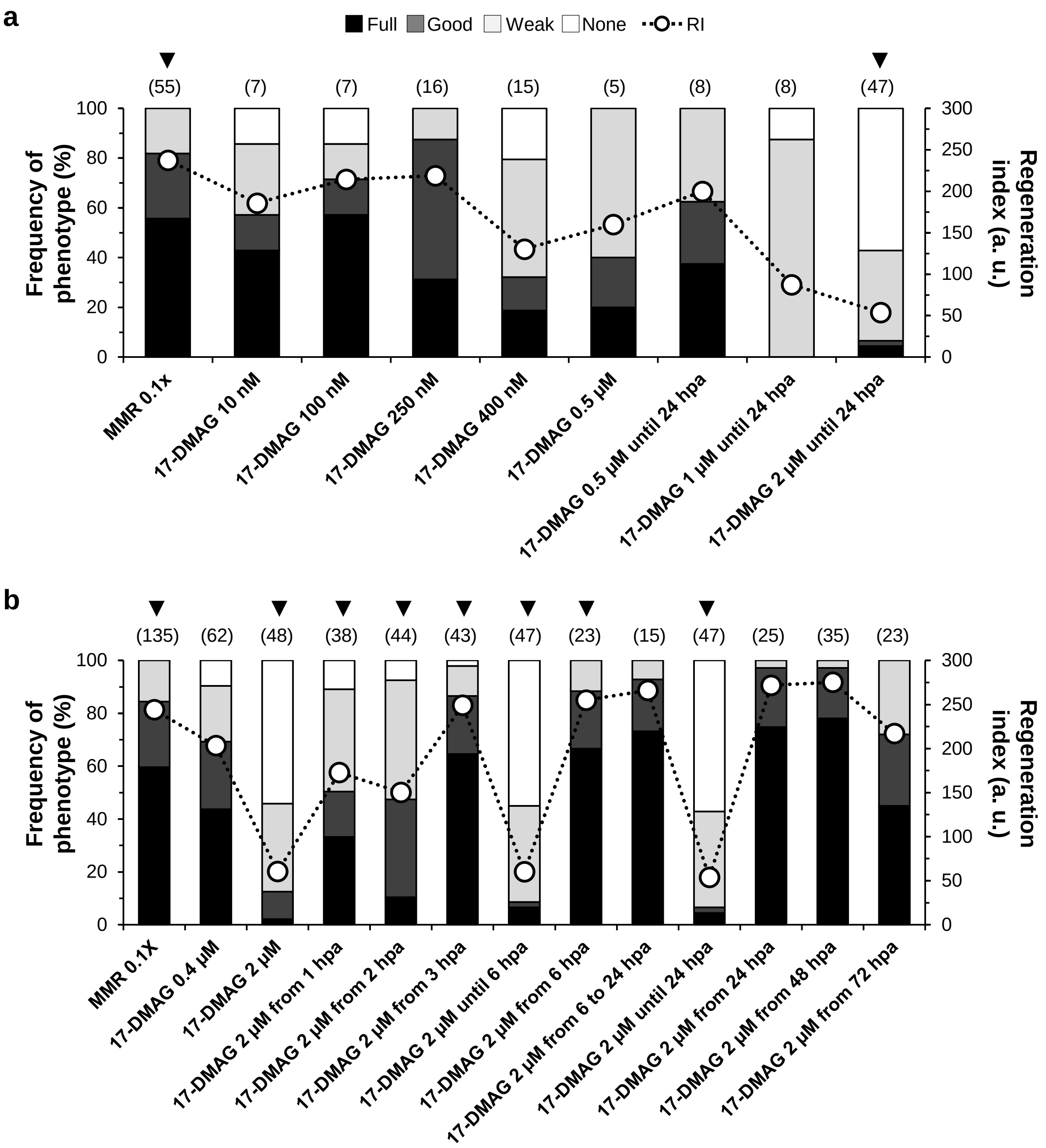

Supplementary Figure 17 | Dose-exposure screening to fine-tune pharmacological treatments during regeneration: 17-DMAG. (**a**) Gross screening. (**b**) Refined screening. Selection was based on the maximal penetrance with minimal observable toxicity and developmental side defects. Black arrowheads: selected dose-exposures and respective control. RI: regeneration index; a. u.: arbitrary units. *n* biological replicates indicated in brackets.

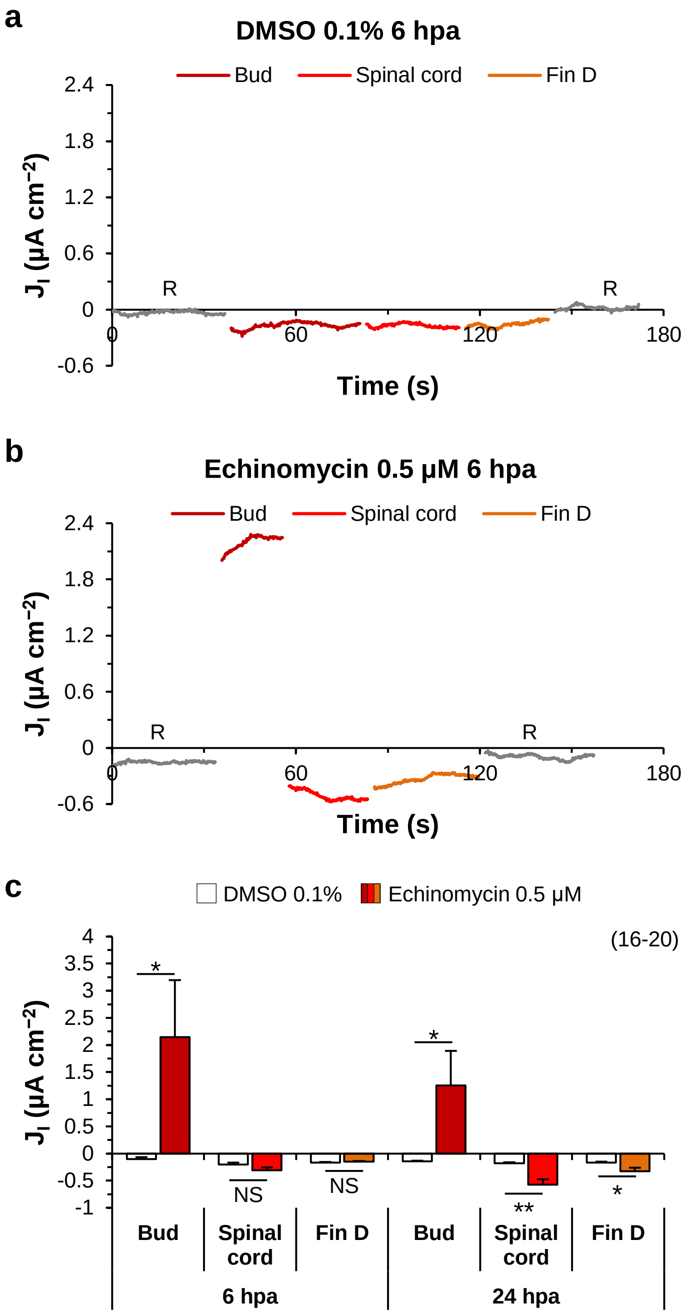

Supplementary Figure 18 | HIF-1α regulates the J_I reversal hallmark in a spatial-dependent way. (**a**,**b**) Representative results. The conventional current flow – direction defined by the flux of positive charge – is used, hence positive values are net outward and negative are net inward currents. Reference (R) and ROI measurements are delimited by a discontinuity in the solid lines. (**c**) Spatial profile of J_I measured in two different time-points in vehicle-control and echinomycin-treated tadpoles amputated at st. 40-41. Data of regeneration bud are the same as those shown in Fig. 7e. Statistical analyses were performed by unpaired Student's *t*-test (two-tailed p value). Data are presented as mean±s.e.m. *n* biological replicates indicated in brackets. NS, non-significant; *, $p<0.05$; **, $p<0.01$.

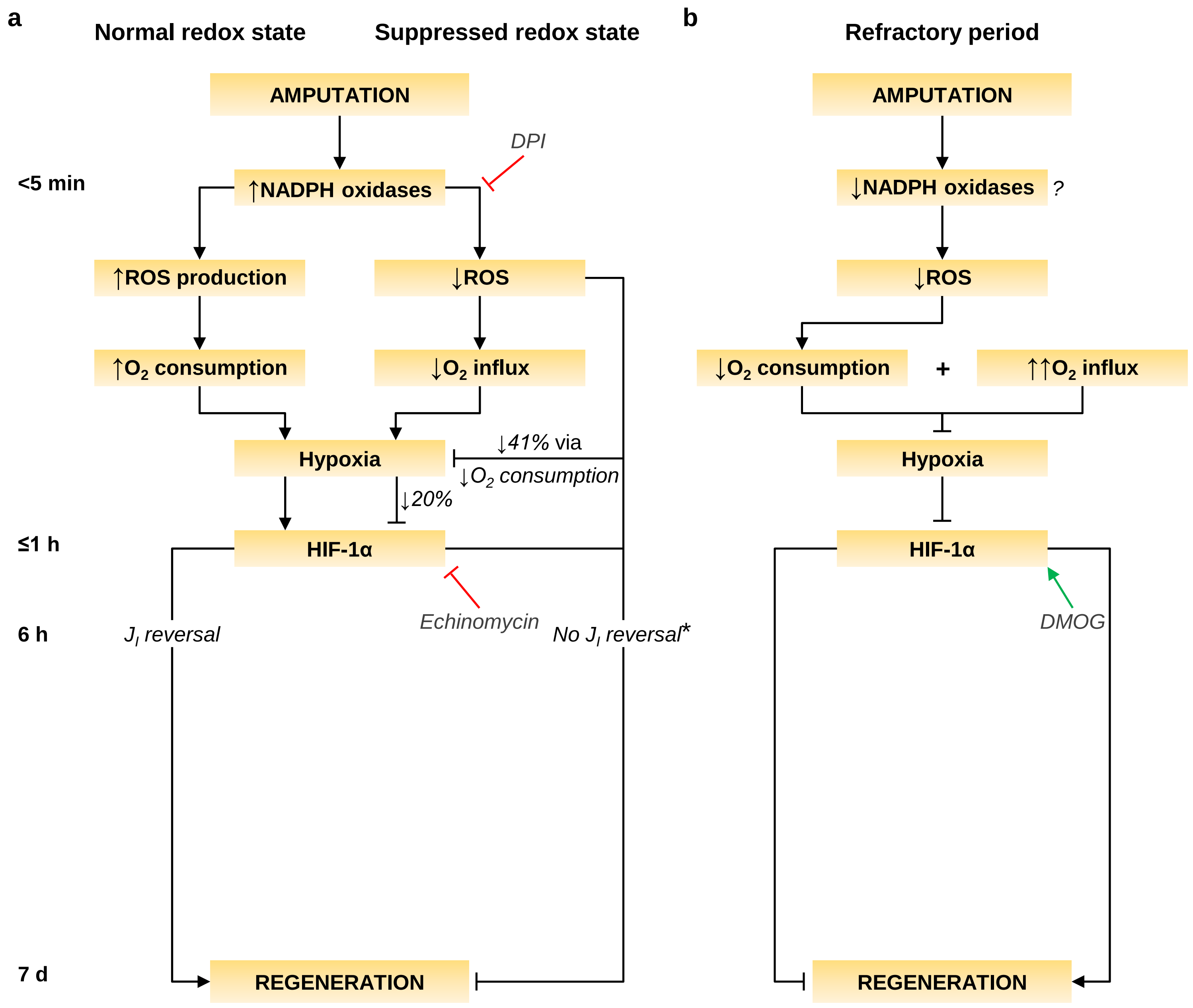

Supplementary Figure 19 | Stepwise model integrating redox state activities during regeneration. (**a**) Early antagonistic ROS production levels generate (and/or contribute to) and sustain considerable local hypoxia. Evidence-based mechanism explaining how either ROS production presence or absence generate (and/or contribute to) hypoxia. In normal redox state (left text boxes), the NADPH oxidase-mediated ROS production consumes O_2 locally, decreasing local pO_2. In suppressed redox state, NADPH oxidase inactivity demands less O_2 influx, decreasing local pO_2. Other hypoxia-generating sources, *e.g.*, vasculature disruption, are not excluded and are probably cumulative. Downstream hypoxia stabilizes HIF-1α that modulates regeneration *via* J_I reversal. However, ROS also modulates J_I reversal, impairing regeneration through this pathway, despite the presence of considerable hypoxia and HIF-1α stability (Fig. 5a-e). HIF-1α and ROS might regulate J_I reversal in series or in parallel. *: ROS effect on J_I reversal demonstrated in[5]. (**b**) Tadpoles amputated in the refractory period produce low levels of ROS (likely due to lower NADPH oxidases activity), therefore consuming less O_2 than

regenerative tadpoles. In addition, these tails have a larger O_2 influx. Together, low consumption and high influx, increases local pO_2, disrupting hypoxia (Fig. 5a). Without hypoxia, HIF-1α undergoes degradation, impairing regeneration. DMOG mimics hypoxia, stabilizing HIF-1α that, in turn, induces regeneration. Timing in **a** applies to **b**. Italic dark grey font: drugs; green closed arrow: pharmacological activation; red bar arrow: pharmacological inhibition.

Supplementary References

1. Nieuwkoop, P. D. & Faber, J. *Normal table of Xenopus laevis (Daudin)*. (Amsterdam: North-Holland, 1967).
2. Beck, C. W., Christen, B. & Slack, J. M. W. Molecular pathways needed for regeneration of spinal cord and muscle in a vertebrate. *Dev. Cell* **5,** 429–39 (2003).
3. Reid, B., Song, B. & Zhao, M. Electric currents in Xenopus tadpole tail regeneration. *Dev. Biol.* **335,** 198–207 (2009).
4. Tseng, A.-S., Beane, W. S., Lemire, J. M., Masi, A. & Levin, M. Induction of vertebrate regeneration by a transient sodium current. *J. Neurosci.* **30,** 13192–200 (2010).
5. Ferreira, F., Luxardi, G., Reid, B. & Zhao, M. Early bioelectric activities mediate redox-modulated regeneration. *Development* **143,** 4582–4594 (2016).
